# Dental sciences related articles data published in a Basic Medical Sciences Journal from Iran

**DOI:** 10.1016/j.dib.2018.02.014

**Published:** 2018-02-13

**Authors:** Thorakkal Shamim

**Affiliations:** Dental Assistant Surgeon, Department of Dentistry, Government Taluk Head Quarters Hospital Malappuram, India

## Abstract

This data aimed to audit the dental sciences related articles published in Iranian Journal of Basic Medical Sciences (IJBMS) from 2007 to 2015 over a 9 year period performed using web-based search. The data were analyzed for topic of dental sciences, type of article, international collaborations, source of funding, number of authors and authorship trends. Out of the total 18 data related to dental sciences, original articles (12), review articles (4) and short communications (2) contribute the major share. Regarding the relationship with dental sciences, the maximum number of data were related to oral pathology and microbiology (16) followed by oral medicine and radiology (7) and periodontics (7). Among the data related to dental sciences, oral cancer (3) and gingival and periodontal diseases (3) followed by dental plaque and caries (2) and orthodontic tooth movement (2) form the major attraction of the contributors. The largest numbers of data related to dental sciences were received from Mashhad University of Medical Sciences, Mashhad (4) and Tehran University of Medical Sciences,Tehran (2).The present data were compared with previous bibliometric studies done related to dental sciences (Shamim et al., 2017a, 2017b)

**Specifications Table**

**Table:** 

Subject area	*Medicine*
More specific subject area	*Dentistry*
Type of data	*Table*
How data was acquired	*web-based search from the journal site http://ijbms.mums.ac.ir/*
Data format	*Descriptive retrospective bibliometric data*
Experimental factors	*Topic of dental sciences, type of article, international collaborations, source of funding, number of authors and authorship trends*
Experimental features	*Bibliometric data*
Data source location	*Iranian Journal of Basic Medical Sciences (IJBMS) from 2007 to 2015 over a 9 year period, Mashhad, Iran*
Data accessibility	*Data are included in the paper*http://ijbms.mums.ac.ir/

**Value of the data**.

These data describe the dental sciences related articles published in a Basic Medical Sciences Journal from Iran.

• These date describe the majority of dental sciences articles published as original articles (12),review articles (4) and short communication (2)
• These data describe specialty wise majority of dental articles related to oral pathology and microbiology (16) followed by oral medicine and radiology (7),periodontics (7), community dentistry (2),orthodontics (2), prosthodontics (1) and oral and maxillofacial surgery (1).
• These data describe topic wise majority of dental articles related to oral cancer (3) and gingival and periodontal diseases (3) followed by dental plaque and caries (2) and orthodontic tooth movement (2)
• The data presented in this paper can be used as a guide for conducting similar studies around the globe.

## 1 Data

See Table 1.

**Table 1:** Dental sciences related articles data published in a Basic Medical Sciences Journal (IJBMS) from Iran from 2007 to 2015

Sl. no	Dental sciences related articles data published in a Basic Medical Sciences Journal (IJBMS) from Iran from 2007 to 2015	Dental speciality-topic-type of article- Institution of the first author- international collaborations -source of funding-number of authors
1	IJBMS Vol 10:2 2007	
1	Amoueian S, Saghafi S, Farhadi F, Tohidi E, Sadegi L. Immunohistochemical assessment of Ki-67 expression in Adenoid cystic carcinoma of the salivary glands. Iran J Basic Med Sci 2007; 10(2):84–89.	Oral pathology and microbiology-salivary gland tumors-original article- Mashhad University of Medical Sciences, Mashhad, Iran –no international collaborations-no source of funding-five
2	IJBMS Vol 10:4 2008	
1	Tahmourespour A, Kermanshahi RK, Salehi R, Nabinejad A. The relationship between cell surface hydrophobicity and antibiotic resistance of Streptococcal strains isolated from dental plaque and caries. Iran J Basic Med Sci 2008; 10(4):251–255.	Periodontics,oral pathology and microbiology-dental plaque and caries –original article - Islamic Azad University Tehran,Iran- no international collaborations-no source of funding-four
3	IJBMS Vol 11:3 2008	
1	Mousavi SH, Tayarani-Najaran Z, Hersey P. Apoptosis: from signalling pathways to therapeutic tools. Iran J Basic Med Sci 2008; 11(3):121–142.	Oral pathology and microbiology,oral medicine and radiology-oral cancer-review article- Mashhad University of Medical Sciences, Mashhad, Iran-international collaborations (Iran,Australia)-no source of funding-three
4	IJBMS Vol 14:2 2011	
1	Hajihoseini S, Bahmani MK, Khosravi A, Ghezelsofla E, Ghaderi A. Prognostic significance of MMP2 and MMP9 functional promoter single nucleotide polymorphisms in head and neck squamous cell carcinoma. Iran J Basic Med Sci 2011; 14(2):137–144.	Oral pathology and microbiology, community dentistry-oral cancer- original article- HIV and Hepatitis Research Center, Faculty of Laboratorial Sciences, Gerash, Fars, Iran- no international collaborations- grant from HIV and Hepatitis Research Center, Shiraz University of Medical Sciences, Shiraz, Iran and partly by a grant from Shiraz Cancer Research Center, Shiraz, Iran-five
2	Saghafi S, Amoueian S, Montazer M, Bostan R. Assessment of VEGF, CD-31 and Ki-67 immunohistochemical markers in oral pyogenic granuloma: A comparison with hemangioma and inflammatory gingivitis. Iran J Basic Med Sci 2011; 14(2):185–189.	Periodontics,oral pathology and microbiology- neoplasms of the oral cavity-short communication- Mashhad University of Medical Sciences, Mashhad, Iran- no international collaborations-grant from Mashhad University of Medical Sciences, Mashhad, Iran- four
5	IJBMS Vol 15:1 2012	
1	Pourkhabbaz A, Pourkhabbaz H. Investigation of toxic metals in the tobacco of different Iranian cigarette brands and related health issues. Iran J Basic Med Sci 2012; 15(1):636–644.	Community dentistry-tobacco control -original article- Birjand University, Birjand, Iran – no international collaborations-grant from Department of Environment Sciences, Birjand University-two
6	IJBMS Vol 15:6 2012	
1	Tahergorabi Z, Khazaei M. A review on angiogenesis and its assays. Iran J Basic Med Sci 2012; 15(6):1110–1126.	Oral pathology and microbiology-oral cancer-review article- Isfahan University of Medical Sciences, Isfahan, Iran- no international collaborations-no source of funding-two
2	Mahdavishahri N, Matin MM, FereidoniM, Yarjanli Z, Rad SAB, Ahmadi SK. in vitro assay of human gingival scaffold in differentiation of rat's bone marrow mesenchymal stem cells to keratinocytes. Iran J Basic Med Sci 2012; 15(6):1185–1190.	Periodontics,oral pathology and microbiology,oral and maxillofacial surgery-oral tissue engineering-original article- Ferdowsi University of Mashhad, Mashhad, Iran- no international collaborations-grant from the Biotechnology Research Centre and the Vice Chancellor of Ferdowsi University of Mashhad, Mashhad, Iran-six
7	IJBMS Vol 16:4 2013	
1	Tahmourespour A, Nabinejad A, Shirian H, Ghasemipero N. The comparison of proteins elaborated by streptococcus mutans strains isolated from caries free and susceptible subjects. Iran J Basic Med Sci 2013; 16(4): 656–660.	Periodontics,oral pathology and microbiology- dental plaque and caries-original article- Islamic Azad University, Isfahan, Iran- no international collaborations-no source of funding- four
8	IJBMS Vol 16:5 2013	
1	Selvi R, Mukunda Priyanka A. Role of Sox9 in the etiology of Pierre-Robin syndrome. Iran J Basic Med Sci 2013; 16(5): 700–704.	Oral pathology and microbiology-cleft lip and cleft palate- original article - Sri Ramachandra University, Tamil Nadu, India – no international collaborations-no source of funding-two
9	IJBMS Vol 16:6 2013	
1	Eteraf-OskoueiT, Najafi M. Traditional and Modern Uses of Natural Honey in Human Diseases: A Review. Iran J Basic Med Sci 2013; 16(6): 731–742.	Periodontitis,oral medicine and radiology-gingival and periodontal disease-review article- Tabriz University of Medical Sciences, Tabriz, Iran- no international collaborations-grant from Research Affairs of Tabriz University of Medical Sciences, Tabriz, Iran- two
10	IJBMS Vol 16:10 2013	
1	Frozanfar A, Ramezani M, Rahpeyma A, Khajeh ahmadi S, Arbab HR. The effects of low level laser therapy on the expression of collagen type I gene and proliferation of human gingival fibroblasts (Hgf3-Pi 53): in vitro study. Iran J Basic Med Sci 2013; 16(10): 1071–1074.	Periodontics,oral pathology and microbiology-gingival and periodontal diseases –original article- Mashhad University of Medical Sciences, Mashhad, Iran- no international collaborations-grant from the Vice Chancellor for Research of Mashhad University of Medical Sciences, Mashhad, Iran-five
11	IJBMS Vol 17:2 2014	
1	Araghizadeh N, Paknejad M, Alaeddini M, Minaii B, Abdollahi M, Khorasanie R. The efficacy and prophylactic characteristics of omega-3 fatty acids in experimental gingivitis in rats. Iran J Basic Med Sci 2014; 17(2):87–92.	Periodontics,oral medicine and radiology,oral pathology and microbiology-gingival and periodontal diseases–original article- Tehran University of Medical Sciences, Tehran, Iran- no international collaborations-grant fromTehran University of Medical Sciences Health Services-six
12	IJBMS Vol 17:6 2014	
1	Shirazi M, Alimoradi H, Kheirandish Y, Etemad‐Moghadam S, Alaeddini M, Meysamie A, Fatahi Meybodi SAR, Dehpour AR. Pantoprazole, a proton pump inhibitor, increases orthodontic tooth movement in rats. Iran J Basic Med Sci 2014; 17(6):448–453.	Orthodontics,oral medicine and radiology,oral pathology and microbiology-orthodontic tooth movement-original article- Tehran University of Medical Sciences, Tehran, Iran- international collaborations (Iran,New Zealand)-no source of funding-eight
13	IJBMS Vol 17:11 2014	
1	Ashrafi Tamai I, Zahraei Salehi T, Sharifzadeh A, Shokri H, Khosravi AR. Repetitive sequences based on genotyping of Candida albicans isolates obtained from Iranian patients with human immunodeficiency virus. Iran J Basic Med Sci 2014; 17(11):831–835.	Oral pathology and microbiology,oral medicine and radiology-candida albicans -original article- Bu Ali Sina University, Hamedan, Iran- no international collaborations-grant from Research Council of the University of Tehran-five
14	IJBMS Vol 18:6 2015	
1	Garg K, Chandra Sh, Raj V, Fareed W, Zafar M. Molecular and genetic aspects of odontogenic tumors: a review. Iran J Basic Med Sci 2015; 18(6):529‐536.	Oral pathology and microbiology,oral medicine and radiology-odontogenic tumors-review article- Chandra Dental College, Barabanki, India-international collaborations (India and Saudi Arabia)- no source of funding-five
15	IJBMS Vol 18:9 2015	
1	Zhang Y, Wang X, Zhang M, Lin X, Wu Q, Yang Y, Kong J, Ji P. The trophic effect of ciliary neurotrophic factor on injured masseter muscle in rat. Iran J Basic Med Sci 2015; 18(9):920‐926.	Oral pathology and microbiology, Prosthodontics-occlusal trauma–original article- Shandong University, Jinan City, China –no international collaborations- grant from science and technology development plans of Shandong province and Specialized Research Fund for the Doctoral Program of Higher Education- eight
16	IJBMS Vol 18:10 2015	
1	Qun Xiao L, Tao Wang H, Lan Li Y, Zeng Q, Zhou E, Ni X, Ping Huan Zh. The effects of dried root aqueous extract of Salvia miltiorrhiza and its major ingredient in acceleration of orthodontic tooth movement in rat. Iran J Basic Med Sci 2015; 18(10):1044‐1049.	Orthodontics,oral medicine and radiology,oral pathology and microbiology-orthodontic tooth movement –short communication- Three Gorges University, Yichang, China –no international collaborations-no source of funding-seven

## 2 Materials and methods

Bibliometric information about dental sciences articles data in a clinical medical journal from Iran and ayurveda journals from India is evident in the literature (Shamim et al., 2017a, 2017b) [1, 2]. A total of 62 issues of IJBMS from 2007 to 2015 were analyzed. This was available on the following journal website: http://ijbms.mums.ac.ir/ (last accessed January 16, 2018).

The data published were analyzed for topic of dental sciences, type of article, international collaborations, source of funding, number of authors and authorship trends.The data regarding articles in press (articles that are not yet assigned to an issue) in IJBMS were excluded. The contents of the data were grouped into seven individual dental sciences specialities such oral pathology and microbiology, oral medicine and radiology, oral and maxillofacial surgery, periodontics, prosthodontics, orthodontics and community dentistry. The data was scrutinized for dental sciences related topic. The datas which are interdisciplinary in approach were counted under all the dental sciences specialities it belonged. The data were checked for authorship trends according to the institution of the first author.The data was analyzed using descriptive statistics.
